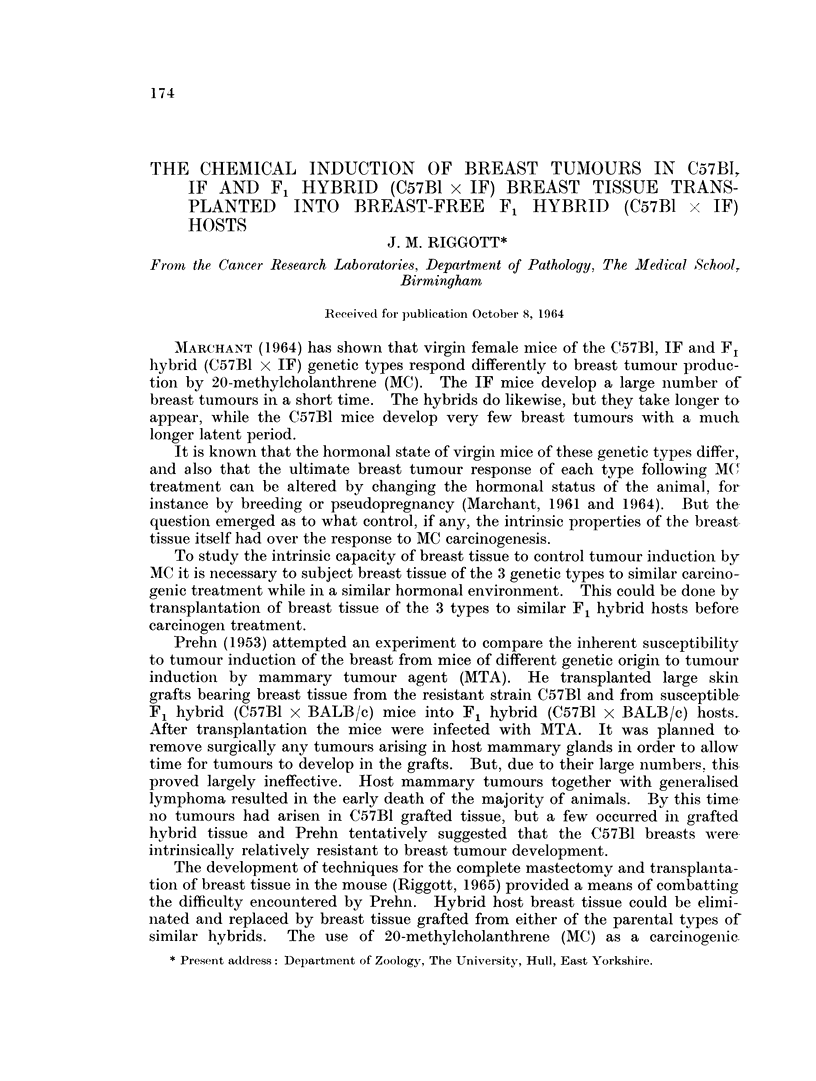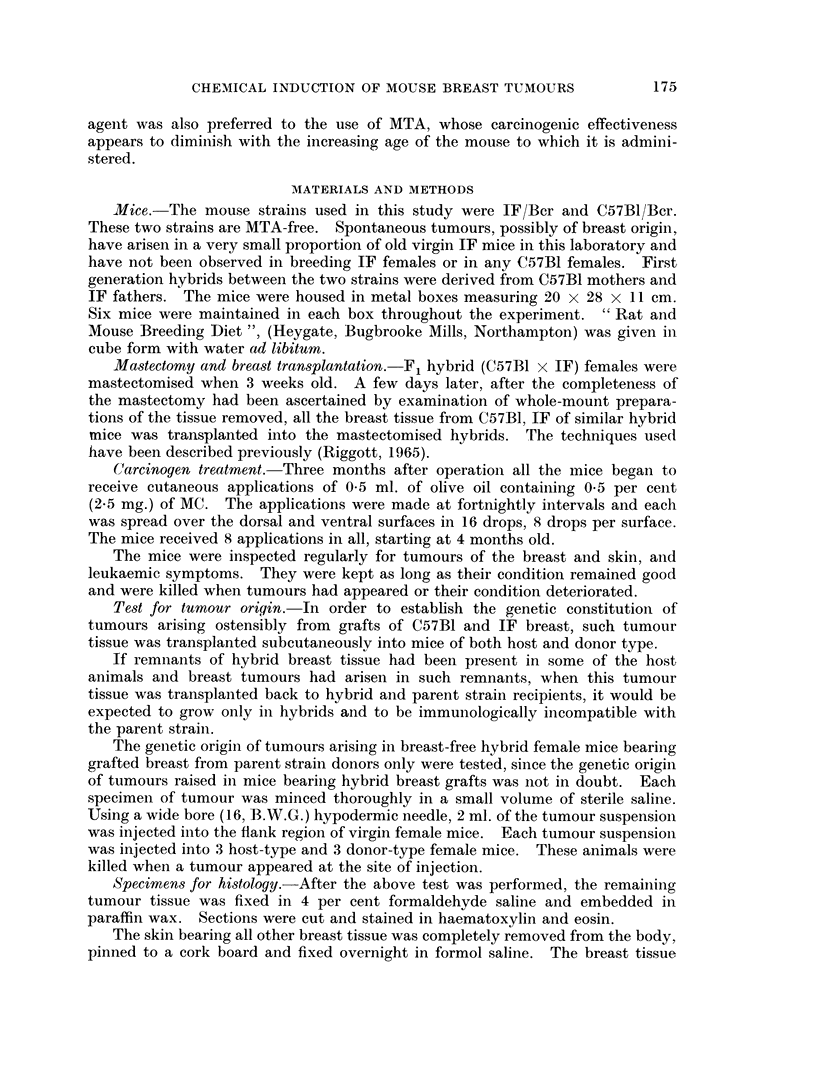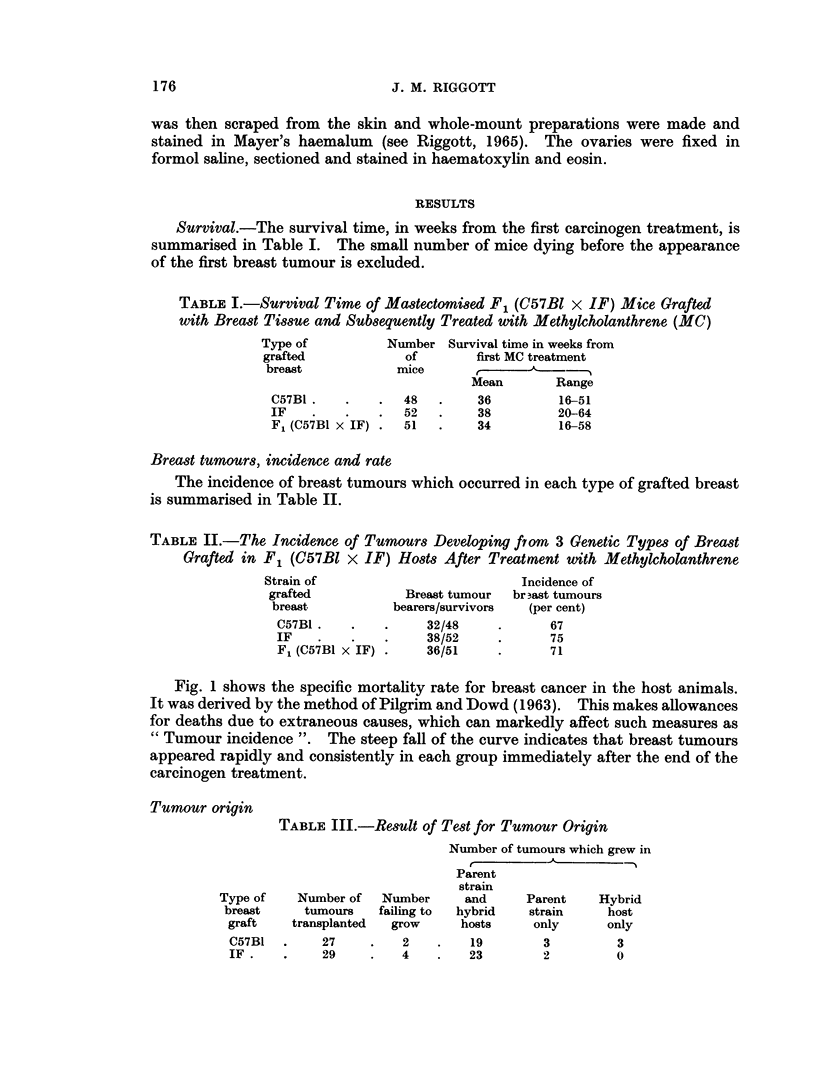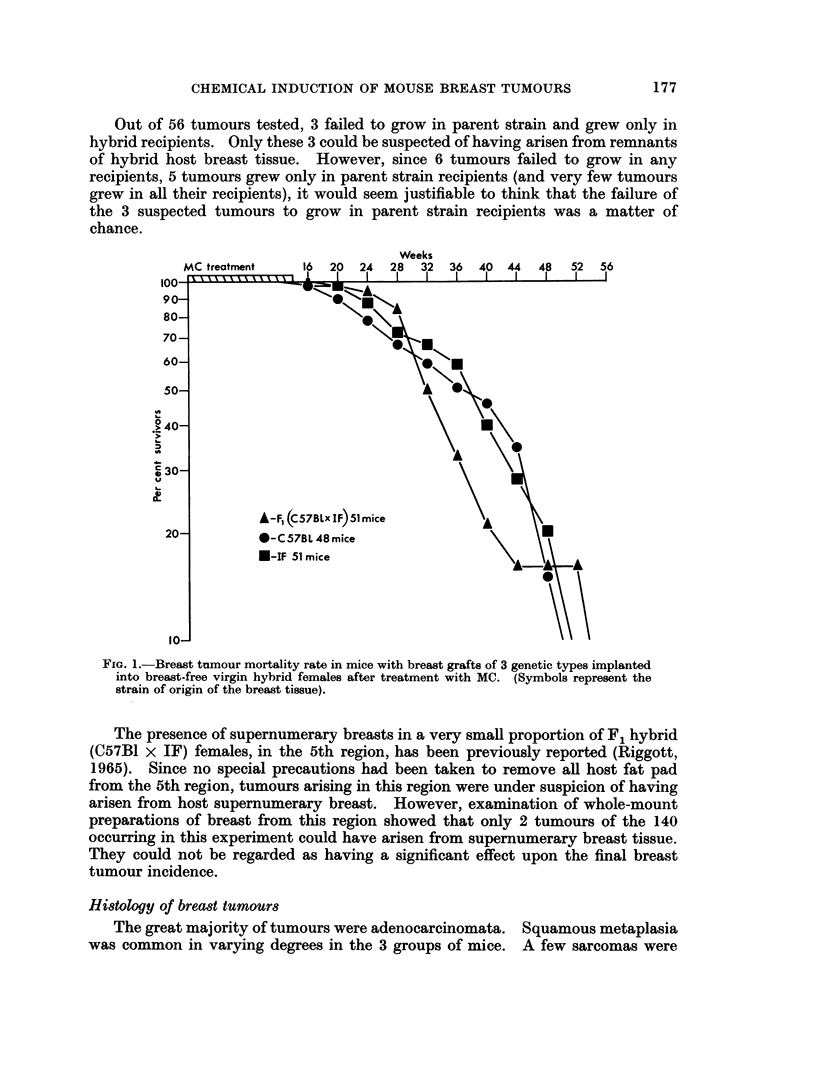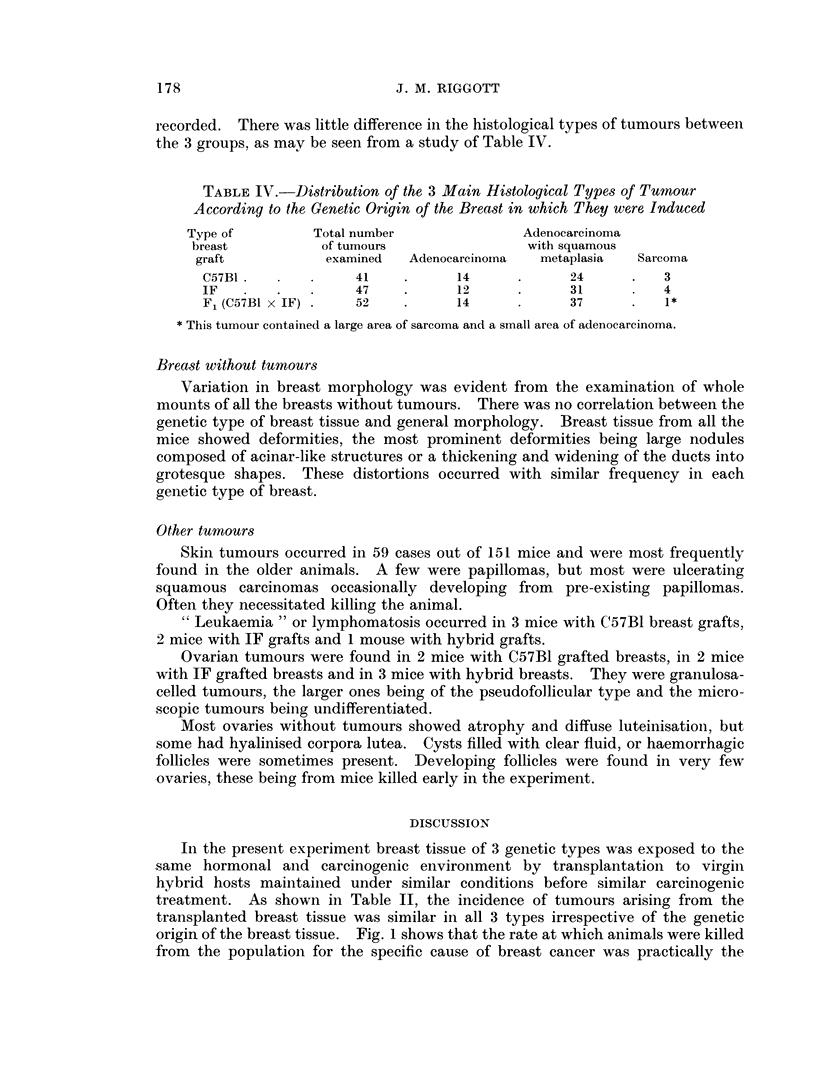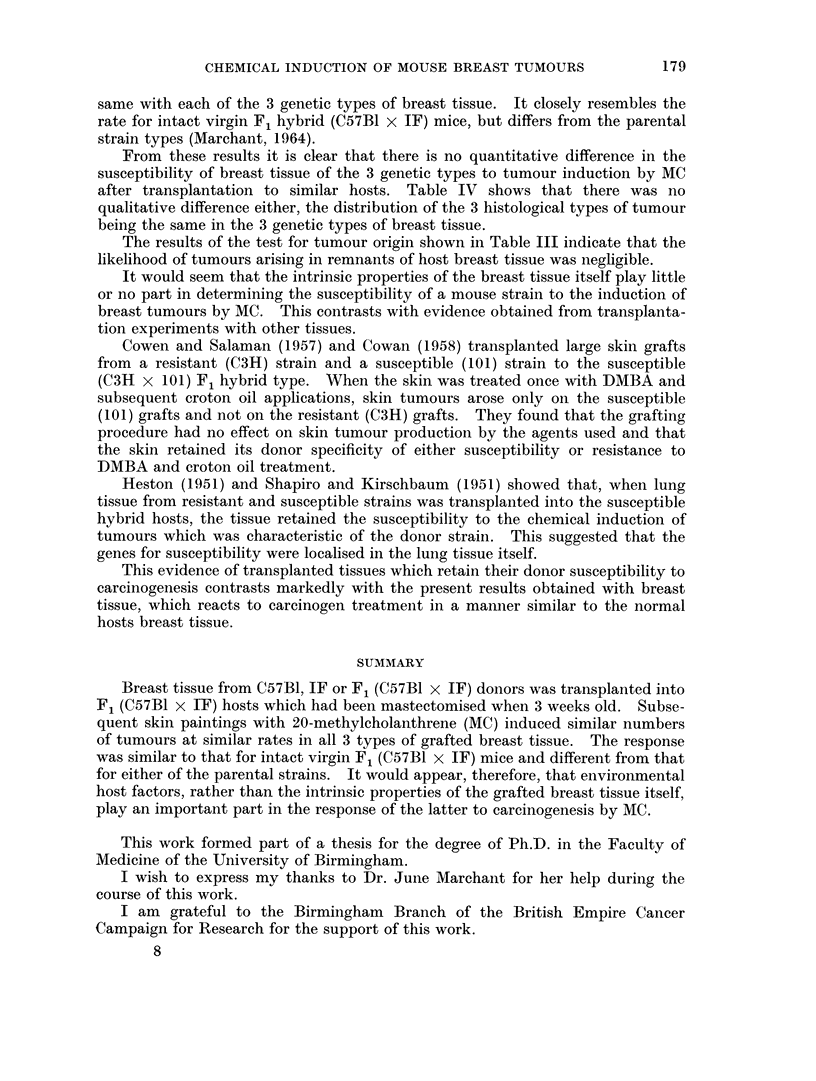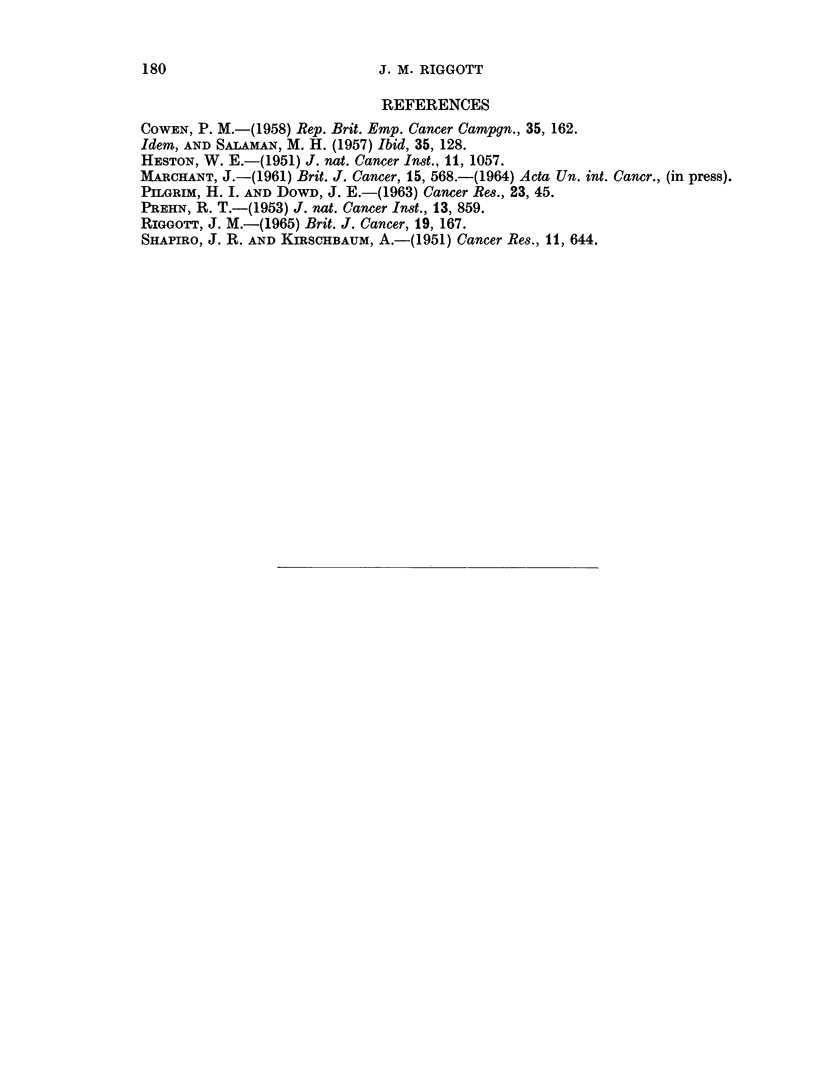# The Chemical Induction of Breast Tumours in C57B1, IF and F_1_ Hybrid (C57B1×IF) Breast Tissue Transplanted into Breast Free F_1_ Hybrid (C57B1 × IF) Hosts

**DOI:** 10.1038/bjc.1965.21

**Published:** 1965-03

**Authors:** J. M. Riggott


### THE CHEMICAL INDUCTION OF BREAST TUMOURS IN C57Bl, IF AND $F_1$ HYBRID (C57Bl × IF) BREAST TISSUE TRANSPLANTED INTO BREAST-FREE $F_1$ HYBRID (C57Bl × IF) HOSTS

J. M. RIGGOTT*

*From the Cancer Research Laboratories, Department of Pathology, The Medical School, Birmingham*

Received for publication October 8, 1964

MARCHANT (1964) has shown that virgin female mice of the C57Bl, IF and $F_1$ hybrid (C57Bl × IF) genetic types respond differently to breast tumour production by 20-methylcholanthrene (MC). The IF mice develop a large number of breast tumours in a short time. The hybrids do likewise, but they take longer to appear, while the C57Bl mice develop very few breast tumours with a much longer latent period.

It is known that the hormonal state of virgin mice of these genetic types differ, and also that the ultimate breast tumour response of each type following MC treatment can be altered by changing the hormonal status of the animal, for instance by breeding or pseudopregnancy (Marchant, 1961 and 1964). But the question emerged as to what control, if any, the intrinsic properties of the breast tissue itself had over the response to MC carcinogenesis.

To study the intrinsic capacity of breast tissue to control tumour induction by MC it is necessary to subject breast tissue of the 3 genetic types to similar carcinogenic treatment while in a similar hormonal environment. This could be done by transplantation of breast tissue of the 3 types to similar $F_1$ hybrid hosts before carcinogen treatment.

Prehn (1953) attempted an experiment to compare the inherent susceptibility to tumour induction of the breast from mice of different genetic origin to tumour induction by mammary tumour agent (MTA). He transplanted large skin grafts bearing breast tissue from the resistant strain C57Bl and from susceptible $F_1$ hybrid (C57Bl × BALB/c) mice into $F_1$ hybrid (C57Bl × BALB/c) hosts. After transplantation the mice were infected with MTA. It was planned to remove surgically any tumours arising in host mammary glands in order to allow time for tumours to develop in the grafts. But, due to their large numbers, this proved largely ineffective. Host mammary tumours together with generalised lymphoma resulted in the early death of the majority of animals. By this time no tumours had arisen in C57Bl grafted tissue, but a few occurred in grafted hybrid tissue and Prehn tentatively suggested that the C57Bl breasts were intrinsically relatively resistant to breast tumour development.

The development of techniques for the complete mastectomy and transplantation of breast tissue in the mouse (Riggott, 1965) provided a means of combatting the difficulty encountered by Prehn. Hybrid host breast tissue could be eliminated and replaced by breast tissue grafted from either of the parental types of similar hybrids. The use of 20-methylcholanthrene (MC) as a carcinogenic

* Present address: Department of Zoology, The University, Hull, East Yorkshire.

agent was also preferred to the use of MTA, whose carcinogenic effectiveness appears to diminish with the increasing age of the mouse to which it is administered.

### MATERIALS AND METHODS

*Mice.*—The mouse strains used in this study were IF/Bcr and C57Bl/Bcr. These two strains are MTA-free. Spontaneous tumours, possibly of breast origin, have arisen in a very small proportion of old virgin IF mice in this laboratory and have not been observed in breeding IF females or in any C57Bl females. First generation hybrids between the two strains were derived from C57Bl mothers and IF fathers. The mice were housed in metal boxes measuring 20 × 28 × 11 cm. Six mice were maintained in each box throughout the experiment. " Rat and Mouse Breeding Diet ", (Heygate, Bugbrooke Mills, Northampton) was given in cube form with water *ad libitum.*

*Mastectomy and breast transplantation.*—$F_1$ hybrid (C57Bl × IF) females were mastectomised when 3 weeks old. A few days later, after the completeness of the mastectomy had been ascertained by examination of whole-mount preparations of the tissue removed, all the breast tissue from C57Bl, IF of similar hybrid mice was transplanted into the mastectomised hybrids. The techniques used have been described previously (Riggott, 1965).

*Carcinogen treatment.*—Three months after operation all the mice began to receive cutaneous applications of 0·5 ml. of olive oil containing 0·5 per cent (2·5 mg.) of MC. The applications were made at fortnightly intervals and each was spread over the dorsal and ventral surfaces in 16 drops, 8 drops per surface. The mice received 8 applications in all, starting at 4 months old.

The mice were inspected regularly for tumours of the breast and skin, and leukaemic symptoms. They were kept as long as their condition remained good and were killed when tumours had appeared or their condition deteriorated.

*Test for tumour origin.*—In order to establish the genetic constitution of tumours arising ostensibly from grafts of C57Bl and IF breast, such tumour tissue was transplanted subcutaneously into mice of both host and donor type.

If remnants of hybrid breast tissue had been present in some of the host animals and breast tumours had arisen in such remnants, when this tumour tissue was transplanted back to hybrid and parent strain recipients, it would be expected to grow only in hybrids and to be immunologically incompatible with the parent strain.

The genetic origin of tumours arising in breast-free hybrid female mice bearing grafted breast from parent strain donors only were tested, since the genetic origin of tumours raised in mice bearing hybrid breast grafts was not in doubt. Each specimen of tumour was minced thoroughly in a small volume of sterile saline. Using a wide bore (16, B.W.G.) hypodermic needle, 2 ml. of the tumour suspension was injected into the flank region of virgin female mice. Each tumour suspension was injected into 3 host-type and 3 donor-type female mice. These animals were killed when a tumour appeared at the site of injection.

*Specimens for histology.*—After the above test was performed, the remaining tumour tissue was fixed in 4 per cent formaldehyde saline and embedded in paraffin wax. Sections were cut and stained in haematoxylin and eosin.

The skin bearing all other breast tissue was completely removed from the body, pinned to a cork board and fixed overnight in formol saline. The breast tissue was then scraped from the skin and whole-mount preparations were made and stained in Mayer's haemalum (see Riggott, 1965). The ovaries were fixed in formol saline, sectioned and stained in haematoxylin and eosin.

### RESULTS

*Survival.*—The survival time, in weeks from the first carcinogen treatment, is summarised in Table I. The small number of mice dying before the appearance of the first breast tumour is excluded.

TABLE I.—*Survival Time of Mastectomised $F_1$ (C57Bl × IF) Mice Grafted with Breast Tissue and Subsequently Treated with Methylcholanthrene (MC)*

| Type of grafted breast | Number of mice | Survival time in weeks from first MC treatment | |
|---|---|---|---|
| | | Mean | Range |
| C57Bl | 48 | 36 | 16–51 |
| IF | 52 | 38 | 20–64 |
| $F_1$ (C57Bl × IF) | 51 | 34 | 16–58 |

*Breast tumours, incidence and rate*

The incidence of breast tumours which occurred in each type of grafted breast is summarised in Table II.

TABLE II.—*The Incidence of Tumours Developing from 3 Genetic Types of Breast Grafted in $F_1$ (C57Bl × IF) Hosts After Treatment with Methylcholanthrene*

| Strain of grafted breast | Breast tumour bearers/survivors | Incidence of breast tumours (per cent) |
|---|---|---|
| C57Bl | 32/48 | 67 |
| IF | 38/52 | 75 |
| $F_1$ (C57Bl × IF) | 36/51 | 71 |

Fig. 1 shows the specific mortality rate for breast cancer in the host animals. It was derived by the method of Pilgrim and Dowd (1963). This makes allowances for deaths due to extraneous causes, which can markedly affect such measures as " Tumour incidence ". The steep fall of the curve indicates that breast tumours appeared rapidly and consistently in each group immediately after the end of the carcinogen treatment.

*Tumour origin*

TABLE III.—*Result of Test for Tumour Origin*

| Type of breast graft | Number of tumours transplanted | Number failing to grow | Number of tumours which grew in: Parent strain and hybrid hosts | Parent strain only | Hybrid host only |
|---|---|---|---|---|---|
| C57Bl | 27 | 2 | 19 | 3 | 3 |
| IF | 29 | 4 | 23 | 2 | 0 |

Out of 56 tumours tested, 3 failed to grow in parent strain and grew only in hybrid recipients. Only these 3 could be suspected of having arisen from remnants of hybrid host breast tissue. However, since 6 tumours failed to grow in any recipients, 5 tumours grew only in parent strain recipients (and very few tumours grew in all their recipients), it would seem justifiable to think that the failure of the 3 suspected tumours to grow in parent strain recipients was a matter of chance.

FIG. 1.—Breast tumour mortality rate in mice with breast grafts of 3 genetic types implanted into breast-free virgin hybrid females after treatment with MC. (Symbols represent the strain of origin of the breast tissue).

The presence of supernumerary breasts in a very small proportion of $F_1$ hybrid (C57Bl × IF) females, in the 5th region, has been previously reported (Riggott, 1965). Since no special precautions had been taken to remove all host fat pad from the 5th region, tumours arising in this region were under suspicion of having arisen from host supernumerary breast. However, examination of whole-mount preparations of breast from this region showed that only 2 tumours of the 140 occurring in this experiment could have arisen from supernumerary breast tissue. They could not be regarded as having a significant effect upon the final breast tumour incidence.

*Histology of breast tumours*

The great majority of tumours were adenocarcinomata. Squamous metaplasia was common in varying degrees in the 3 groups of mice. A few sarcomas were recorded. There was little difference in the histological types of tumours between the 3 groups, as may be seen from a study of Table IV.

TABLE IV.—*Distribution of the 3 Main Histological Types of Tumour According to the Genetic Origin of the Breast in which They were Induced*

| Type of breast graft | Total number of tumours examined | Adenocarcinoma | Adenocarcinoma with squamous metaplasia | Sarcoma |
|---|---|---|---|---|
| C57Bl . . . | 41 | 14 | 24 | 3 |
| IF . . . | 47 | 12 | 31 | 4 |
| $F_1$ (C57Bl × IF) . | 52 | 14 | 37 | 1* |

* This tumour contained a large area of sarcoma and a small area of adenocarcinoma.

*Breast without tumours*

Variation in breast morphology was evident from the examination of whole mounts of all the breasts without tumours. There was no correlation between the genetic type of breast tissue and general morphology. Breast tissue from all the mice showed deformities, the most prominent deformities being large nodules composed of acinar-like structures or a thickening and widening of the ducts into grotesque shapes. These distortions occurred with similar frequency in each genetic type of breast.

*Other tumours*

Skin tumours occurred in 59 cases out of 151 mice and were most frequently found in the older animals. A few were papillomas, but most were ulcerating squamous carcinomas occasionally developing from pre-existing papillomas. Often they necessitated killing the animal.

" Leukaemia " or lymphomatosis occurred in 3 mice with C57Bl breast grafts, 2 mice with IF grafts and 1 mouse with hybrid grafts.

Ovarian tumours were found in 2 mice with C57Bl grafted breasts, in 2 mice with IF grafted breasts and in 3 mice with hybrid breasts. They were granulosa-celled tumours, the larger ones being of the pseudofollicular type and the microscopic tumours being undifferentiated.

Most ovaries without tumours showed atrophy and diffuse luteinisation, but some had hyalinised corpora lutea. Cysts filled with clear fluid, or haemorrhagic follicles were sometimes present. Developing follicles were found in very few ovaries, these being from mice killed early in the experiment.

### DISCUSSION

In the present experiment breast tissue of 3 genetic types was exposed to the same hormonal and carcinogenic environment by transplantation to virgin hybrid hosts maintained under similar conditions before similar carcinogenic treatment. As shown in Table II, the incidence of tumours arising from the transplanted breast tissue was similar in all 3 types irrespective of the genetic origin of the breast tissue. Fig. 1 shows that the rate at which animals were killed from the population for the specific cause of breast cancer was practically the same with each of the 3 genetic types of breast tissue. It closely resembles the rate for intact virgin $F_1$ hybrid (C57Bl $\times$ IF) mice, but differs from the parental strain types (Marchant, 1964).

From these results it is clear that there is no quantitative difference in the susceptibility of breast tissue of the 3 genetic types to tumour induction by MC after transplantation to similar hosts. Table IV shows that there was no qualitative difference either, the distribution of the 3 histological types of tumour being the same in the 3 genetic types of breast tissue.

The results of the test for tumour origin shown in Table III indicate that the likelihood of tumours arising in remnants of host breast tissue was negligible.

It would seem that the intrinsic properties of the breast tissue itself play little or no part in determining the susceptibility of a mouse strain to the induction of breast tumours by MC. This contrasts with evidence obtained from transplantation experiments with other tissues.

Cowen and Salaman (1957) and Cowan (1958) transplanted large skin grafts from a resistant (C3H) strain and a susceptible (101) strain to the susceptible (C3H $\times$ 101) $F_1$ hybrid type. When the skin was treated once with DMBA and subsequent croton oil applications, skin tumours arose only on the susceptible (101) grafts and not on the resistant (C3H) grafts. They found that the grafting procedure had no effect on skin tumour production by the agents used and that the skin retained its donor specificity of either susceptibility or resistance to DMBA and croton oil treatment.

Heston (1951) and Shapiro and Kirschbaum (1951) showed that, when lung tissue from resistant and susceptible strains was transplanted into the susceptible hybrid hosts, the tissue retained the susceptibility to the chemical induction of tumours which was characteristic of the donor strain. This suggested that the genes for susceptibility were localised in the lung tissue itself.

This evidence of transplanted tissues which retain their donor susceptibility to carcinogenesis contrasts markedly with the present results obtained with breast tissue, which reacts to carcinogen treatment in a manner similar to the normal hosts breast tissue.

### SUMMARY

Breast tissue from C57Bl, IF or $F_1$ (C57Bl $\times$ IF) donors was transplanted into $F_1$ (C57Bl $\times$ IF) hosts which had been mastectomised when 3 weeks old. Subsequent skin paintings with 20-methylcholanthrene (MC) induced similar numbers of tumours at similar rates in all 3 types of grafted breast tissue. The response was similar to that for intact virgin $F_1$ (C57Bl $\times$ IF) mice and different from that for either of the parental strains. It would appear, therefore, that environmental host factors, rather than the intrinsic properties of the grafted breast tissue itself, play an important part in the response of the latter to carcinogenesis by MC.

This work formed part of a thesis for the degree of Ph.D. in the Faculty of Medicine of the University of Birmingham.

I wish to express my thanks to Dr. June Marchant for her help during the course of this work.

I am grateful to the Birmingham Branch of the British Empire Cancer Campaign for Research for the support of this work.

### REFERENCES

Cowen, P. M.—(1958) *Rep. Brit. Emp. Cancer Campgn.*, **35**, 162.
*Idem*, and Salaman, M. H. (1957) *Ibid*, **35**, 128.
Heston, W. E.—(1951) *J. nat. Cancer Inst.*, **11**, 1057.
Marchant, J.—(1961) *Brit. J. Cancer*, **15**, 568.—(1964) *Acta Un. int. Cancr.*, (in press).
Pilgrim, H. I. and Dowd, J. E.—(1963) *Cancer Res.*, **23**, 45.
Prehn, R. T.—(1953) *J. nat. Cancer Inst.*, **13**, 859.
Riggott, J. M.—(1965) *Brit. J. Cancer*, **19**, 167.
Shapiro, J. R. and Kirschbaum, A.—(1951) *Cancer Res.*, **11**, 644.